# Advanced Characterization of FeNi-Based Films for the Development of Magnetic Field Sensors with Tailored Functional Parameters

**DOI:** 10.3390/s22093324

**Published:** 2022-04-26

**Authors:** Sergey V. Komogortsev, Irina G. Vazhenina, Sofya A. Kleshnina, Rauf S. Iskhakov, Vladimir N. Lepalovskij, Anna A. Pasynkova, Andrey V. Svalov

**Affiliations:** 1 Kirensky Institute of Physics, Federal Research Center KSC SB RAS, 660036 Krasnoyarsk, Russia; irina-vazhenina@mail.ru (I.G.V.); sofya.antipckina@yandex.ru (S.A.K.); rauf@iph.krasn.ru (R.S.I.); 2 Institute of Physics, Siberian Federal University, 660041 Krasnoyarsk, Russia; 3 Department of Magnetism of Solid State, Institute of Natural Sciences and Mathematics, Ural Federal University, 620002 Ekaterinburg, Russia; vladimir.lepalovsky@urfu.ru (V.N.L.); pasynkova_a@imp.uran.ru (A.A.P.); andrey.svalov@urfu.ru (A.V.S.); 4 Laboratory of Advanced Magnetic Materials, Institute of Metal Physics UD RAS, 620108 Ekaterinburg, Russia

**Keywords:** magnetic field sensors, thin films, multilayered structures, magnetic anisotropy, anisotropy distribution, ferromagnetic resonance, magnetoimpedance, high frequency applications

## Abstract

Magnetometry and ferromagnetic resonance are used to quantitatively study magnetic anisotropy with an easy axis both in the film plane and perpendicular to it. In the study of single-layer and multilayer permalloy films, it is demonstrated that these methods make it possible not only to investigate the average field of perpendicular and in-plane anisotropy, but also to characterize their inhomogeneity. It is shown that the quantitative data from direct integral and local measurements of magnetic anisotropy are consistent with the direct and indirect estimates based on processing of the magnetization curves. The possibility of estimating the perpendicular magnetic anisotropy constant from the width of stripe domains in a film in the transcritical state is demonstrated. The average in-plane magnetic anisotropy field of permalloy films prepared by magnetron sputtering onto a Corning glass is almost unchanged with the thickness of a single-layer film. The inhomogeneity of the perpendicular anisotropy field for a 500 nm film is greater than that for a 100 nm film, and for a multilayer film with a total permalloy thickness of 500 nm, it is greater than that for a homogeneous film of the same thickness.

## 1. Introduction

The functionality of a magnetic sensor is largely determined by magnetic anisotropy or difference in the magnetic response at different directions of the applied field. In a magnetic film sensor, both the magnetic shape anisotropy (characterized by the easy magnetization plane and anisotropy constant equal to $\mu_{0}M_{s}^{2}/2$) and the contribution to the magnetic anisotropy associated with the material of the magnetic film are important. In addition to the shape anisotropy, two major contributions to the macroscopic magnetic anisotropy of the film are made by the in-plane magnetic anisotropy with an easy magnetization axis (EA) in the film plane ($K_{\mathrm{inplane}}\equiv K_{ip}$, $H_{\mathrm{ip}}=2K_{ip}/\mu_{0}M_{s}$, where $M_{s}$ is the saturation magnetization) and out-of-plane magnetic anisotropy ($K_{\mathrm{out}\;\mathrm{of}\;\mathrm{plane}}\equiv K_{op}$, $H_{\mathrm{op}}=2K_{op}/\mu_{0}M_{s}$). Both the in-plane and out-of-plane magnetic anisotropies are usually induced by the deposition of a thin film or a multilayered structure in the presence of a constant magnetic field [1,2], by inclined sputtering [3,4] due to the anisotropic substrate surface [5,6], film texture [7] or due to anisotropic stresses via magnetoelastic phenomena [8].

The $H_{\mathrm{ip}}$ field value is almost the upper limit of fields, which can be detected by the sensor. The values of $K_{op}$ or $H_{\mathrm{op}}$ limit the film thicknesses of the sensitive element components. This limitation is associated with the transition to the “transcritical” state with the thickness L of the uniform film greater than the critical thickness $L_{\mathrm{cr}}=2\pi \sqrt{\left(A/K_{op}\right)}$ (where A is the exchange stiffness). The magnetic hysteresis of the film in this state $\left(L>L_{\mathrm{cr}}\right)$ sharply increases, and the in-plane magnetic anisotropy becomes weakly pronounced due to the appearance of rotatable magnetic anisotropy [9,10]. The transition into the “transcritical” state is accompanied by the formation of stripe domains oriented parallel to the external magnetic field previously applied to the film in the film plane.

The easy magnetization axis of the rotatable anisotropy is parallel to the stripes. The stripe domains can be arranged by a certain applied field in any direction in the film plane, which implies that the easy magnetization axis along the stripes is rotated by the applied field as well [2]. Usually, the rotatable anisotropy in soft magnetic films exceeds the induced magnetic anisotropy, and thus, the total magnetic anisotropy in the film plane becomes weakly pronounced. The high magnetic hysteresis and weakly pronounced magnetic anisotropy strongly decrease the sensitivity of the thin film element with respect to the external magnetic field, making the element unsuitable for the magnetic field sensing. This limitation is sometimes in conflict with the technical requirements for the sensor parameters, in particular, concerning the requirementsfor particular applications (such as GMI) demanding films with a thickness exceeding $L_{\mathrm{cr}}$. An example of resolving such a contradiction is a multilayer design of a magnetic thin film-based element [2]. Magnetic sensors with sensitive elements containing single-layered or multilayered film structures are of interest for applications in electronic devices and biomedicine [11,12,13].

Iron–nickel Fe_20_Ni_80_ (permalloy)-based films are the best historically proven choice for magnetic nanostructured-sensitive elements with high magnetic permeability and fairly stable characteristics in different media, wide temperature ranges and various radiation levels (up to airspace conditions) [14,15,16]. Permalloy-based multilayers are becoming more and more important in sensor design due to a number of advantages, including the developed methodologies to avoid the transition into the “transcritical” state for rather thick films [9,10,17,18], which are required for many high-frequency applications, as well as efficient sensors working on the principle of the giant magnetoimpedance effect (GMI) [14,15,16].

New requirements for the functional properties of thin film-based sensors call for the development of measurement and characterization approaches leading to the deeper understanding both of integral and local properties of thin films and multilayered structures. The approaches traditionally used in magnetic anisotropy characterization (measurement of magnetization curves and ferromagnetic resonance—FMR) are continuously improved, both through the development of standard magnetometers and spectrometers with enhanced sensitivities and resolutions. The development of new approaches to data analysis also contributes to this progress. The latter is important in the research related to the inhomogeneity of magnetic anisotropy (both the variation value of the anisotropy constant K and the variation of the easy magnetization axis direction). The inhomogeneity of the magnetic anisotropy is due both to the variation of the technological parameters during the thin film fabrication and to the natural features of the process of thin film growth. The above-mentioned contributions are quite common for many types of magnetic films and they should be taken into account during the fabrication of the sensitive element of magnetic films. Such an inhomogeneity can narrow the range of linear and reversible responses of the film element. In this work, we discuss magnetic anisotropy measurements using ferromagnetic resonance and magnetometry and compare various approaches to the data processing using thin films and multilayered permalloy-based systems.

In this study, we propose, discuss and develop several approaches for the detailed evaluation of magnetic anisotropy using static and dynamic magnetic measurements, including ferromagnetic resonance, as a tool for the comprehensive characterization of thin films and multilayered permalloy-based structures.

## 2. Experiment

FeNi-based films including single-layer and multilayer films (Fe_20_Ni_80_ (100 nm)/Cu(3 nm))_5_ were prepared by magnetron sputtering onto Corning glass substrates at room temperature. The background pressure was 3 × 10^−7^ mbar, and the working argon pressure was 3 × 10^−3^ mbar. Permalloy films (Py) were deposited using a Fe_20_Ni_80_ alloy target. The thickness control of the layers was carried out through the deposition time based on the previously calibrated deposition rates. The single-layer FeNi film thickness was varied in a range of 50–500 nm. A constant magnetic field of 20 kA/m was applied parallel to the film plane during the deposition in order to induce a well-defined uniaxial magnetic anisotropy. In some cases, a Ta buffer layer was used in order to improve the properties of the FeNi thin films. The use of the Ta buffer layer leads to a more perfect crystal structure of permalloy films, which, in turn, contributes to a decrease in their coercive force [19,20]. The compositions of the obtained FeNi films were determined by energy dispersive X-ray analysis and in all the cases under consideration it was close to the Fe_20_Ni_80_ permalloy composition with the near-to-zero magnetostriction constant [2]. Magnetic hysteresis loops (both in-plain and out of plane) were measured by a vibrating sample magnetometer. The images of domain structures were obtained by the magneto-optical Kerr effect using an optical microscope (Evico, Dresden, Germany).

The microwave absorption spectra were measured using the equipment of the Krasnoyarsk Regional Center of Research Equipment of the Federal Research Center “Krasnoyarsk Science Center SB RAS” (spectrometer ELEXSYS E580, Bruker, Bremen, Germany). The spectra were acquired at room temperature in the X-band (the resonator pumping frequency was f = 9.43 GHz). The sample was placed into an antinode of the oscillating magnetic field h~ of the cavity resonator, and the external constant magnetic field was applied in the film plane.

## 3. Results and Discussion

### 3.1. In-Plane Magnetic Anisotropy

**Figure 1:**
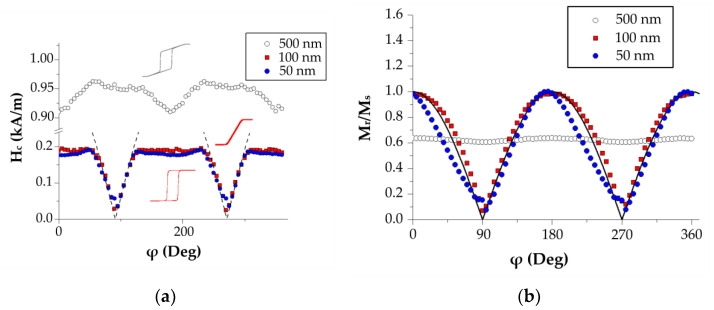
The parameters of the hysteresis loop of permalloy films of various thicknesses: (**a**) coercive force and (**b**) remnant magnetization. The dashed line in (**a**) is the equation $H_{c}(\phi )=H_{ip}\left|\cos(\phi )\right|$; the solid line in (**b**) is $f(\phi )=\left|\cos(\phi )\right|$.

The transition from the thin film state into the “transcritical” state is clearly observed on the hysteresis loops of the single-layer Py/Ta films (Py here and further implies permalloy Fe_20_Ni_80_) with the thicknesses of 50, 100 or 500 nm (Figure 1). This transition results in a sharp increase in the coercive force, change in the loop shape, and almost complete disappearance of the magnetic anisotropy in the film plane. As a consequence of such transition, a perpendicular magnetic anisotropy component appears. Magnetron sputtering of the Py layers is intentionally performed in a field applied in the film plane during the film deposition, which leads to the formation of an in-plane magnetic anisotropy with EA along the direction of the applied field [2]. In Figure 1, this direction corresponds to the angle ϕ=0° between the applied field and the field axis during the deposition.

The formation of EA in the film plane induced under these deposition conditions is confirmed by the shape of the hysteresis loops (Figure 1) for the films with the thicknesses of 50 and 100 nm. The coincidence of the loop shape for the films of 50 and 100 nm means that the film surface does not significantly contribute to the hysteresis. Apparently, it is controlled only by the bulk properties of the film (for example, by the features of the induced magnetic anisotropy). It also means that the hysteresis properties of the Py layer are uniform though the film thickness. The Stoner–Wohlfarth model here well describes the coercivity angular dependence $H_{c}(\phi )$ in the angular range of ±15° on the hard magnetization axis (dashed lines in Figure 1a). At other angles, the $H_{c}(\phi )$ dependence corresponds qualitatively to the inhomogeneous magnetization associated with the nucleation of reverse magnetization domains and motion of the domain walls. Fitting the $H_{c}(\phi )$ data in the range of angles ±15° from the direction corresponding to the hard magnetization axis by the equation $H_{c}(\phi )=H_{ip}\left|\cos(\phi )\right|$ provides an estimate of the magnetic anisotropy field $H_{ip}=0.40\pm 0.08$ kA/m.

**Figure 2:**
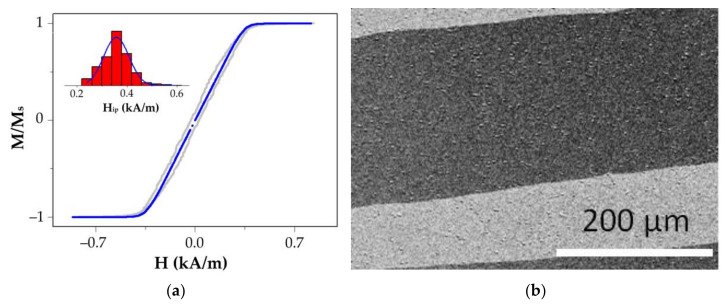
The magnetization curve of the Py (100 nm) film in the in-plane applied magnetic field perpendicular to the in-plane easy magnetization axis (gray symbols) and its fitting using Formula (1) (the inset shows the evaluation result for the film Py (100 nm)) (**a**). The magnetic domain structure in the zero magnetic field; the easy magnetization axis is oriented close to the horizontal direction (**b**).

The saturation field estimation from the magnetic hysteresis loop measured in the hard magnetization direction is the most common approach for estimating the in-plane magnetic anisotropy field. The magnetization curve of the Py (100 nm) film in the in-plane applied magnetic field perpendicular to in-plane easy magnetization axis (gray symbols in Figure 2a) and its fitting using the formula:

(1) $$M(H)=\left\{\begin{array}{c} M_{s}\sum f_{i}\cdot \frac{H}{H_{ip}},\;for\;\left|H\right|<H_{ip}\\ M_{s},\;for\;\left|H\right|>H_{ip} \end{array}\right.$$

where $f_{i}$ is the statistic weight of sites with the specific H_ip_ value, which makes it possible to estimate the inhomogeneity of the anisotropy field $H_{ip}$ (inset in Figure 2a). The Kerr image in Figure 2b shows large stripe domains typical for the subcritical (thin-film) state of the film. Below, we show that the film in the transcritical state (thick-film) shows stripe domains that are two orders of magnitude narrower.

According to the Stoner–Wohlfarth model, the saturation field in the direction perpendicular to the axis of the easiest magnetization is equal to the magnetic anisotropy field. The loop shape within the framework of the model is nearly linear in the range of $-H_{s}\cdot (M=M_{s})$ to $H_{s}\cdot (M=M_{s})$. Outside this range, the sample is uniformly magnetized to the saturation $\left(M=\pm M_{s}\right)$. In the experiment, in a perfectly homogeneous film, a feature near the field $H_{s}=H_{a}$ would be observed as a sharp gap on the M(H) curve, or a discontinuity in the susceptibility χ(H)=dM/dH. There can also be a narrow peak in the field dependence of dχ/dH. Since the inhomogeneity of the anisotropy field somewhat blurs the gap in M(H) near $H_{s}$, this feature is used to quantify the inhomogeneity of the magnetic anisotropy field [21,22]. An estimate of the magnetic anisotropy field inhomogeneity in the film plane is shown in Figure 2 (inset), with the distribution center $H_{ip}=357\pm 5$ A/m, and FWHM (full width at half maximum)=120 A/m.

**Figure 3:**
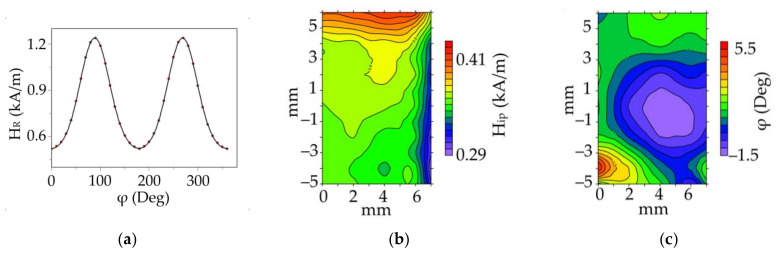
The angular dependence of the resonant field of a pixel in the center of the single-layer film of Py (100 nm) (**a**) Inhomogeneity of the uniaxial magnetic anisotropy field in the plane of thesingle-layer film of Py (100 nm) (**b**) Orientation inhomogeneity of the in-plane easy axis in the plane of the single-layer film of Py (100 nm) (**c**) Deviation from the average in-plane easy axis.

Interesting possibilities for estimating the inhomogeneity of magnetic anisotropy are provided by the measurements of the magnetic parameters of local areas in the sample using an automated scanning FMR spectrometer [8,23], in which a miniature micro-strip resonator fabricated on a substrate with a high dielectric constant is employed as a thin film based sensitive element. Near the antinode of the high-frequency magnetic field, a small measuring hole was made in the resonator screen, ensuring the locality of measurements (for the data in Figure 3 the diameter of the measuring hole of the sensor was ~1 mm). The idea to use the microwave techniques based on a conventional homodyne spectrometer, as a microwave microscope was reported in previous publications [24,25]. Bhagat et al. employed a microwave microscope, i.e., a 2 mm diameter hole in a thinned wall of the cavity for the evaluation of the properties of the FeNi film placed outside the cavity in front of the hole [25]. This methodology has the advantages of avoiding extra-large loading and offers a possibility to estimate the homogeneity of the properties by exposing different regions of the sample to microwaves. However, the system had only a manual displacement mode allowing limited number of points. In addition, it was possible to make measurements from two sides of the film deposited onto a glass substrate: from the side of the film and from the side of the substrate. The equipment described in the present work has an advantage of a scanning system and higher resolution of measurements as the scanning hole had a two times lower diameter of the hole.

In this work, a microwave sensor with a pump frequency f=1.010 GHz was used for these measurements. In Figure 3, we demonstrate the approach to the quantification of the local magnetic anisotropy (Figure 3a) and the result of studying the inhomogeneity of magnetic anisotropy in the plane of the single-layer film of Py (100 nm) (Figure 3b,c). For the given frequency with the applied field in the film plane, the resonant fields in the single-layer film of Py (100 nm) did not exceed 1.6 kA/m. The angular dependences of the resonant field $H_{R}$ were recorded in each local area with a step of 1 mm over the entire surface of the film. Then, the main magnetic characteristics of the films were determined from these data on the basis of a phenomenological calculation [23,26]. The obtainment of the anisotropy constant from the angular dependence of the resonant field is based on the dependence of the resonant field on the equilibrium position of the magnetization, which is described by the Stoner–Wohlfarth model (Smit–Beljers approach [26]). When the external magnetic field is applied in the film plane, in this model, the in-plane anisotropy field is the only important parameter. We used the software developed in [23], which has additional options to quantify not only the uniaxial constants, but also higher-order anisotropy contributions. The major contribution to the angular dependence of the resonant field comes from the uniaxial in-plane anisotropy. The maps of the in-plane anisotropy field and orientation of EA (Figure 3) show the local value of the in plane magnetic anisotropy. The inhomogeneity of the anisotropy field along the right and upper edges of the sample (Figure 3) is due to deformations that occur in the process of cutting the sample. A more uniform distribution is observed along the lower and left edges of the sample that are not subjected to the cut process. The uniformity in the anisotropy field is satisfactory and the inhomogeneity in the orientation of the EA over an area of 5×7 mm does not exceed 6÷7°. The average field obtained here $H_{ip}=340\pm 20$ A/m is close to the anisotropy field estimated from the hysteresis loops. It is important that direct local measurements of the magnetic anisotropy (Figure 3b,c) provide not only an estimate of the inhomogeneity of magnetic anisotropy, but also visualize the spatial pattern of the distribution of the local magnetic anisotropy.

### 3.2. Out-of-Plane Magnetic Anisotropy

**Figure 4:**
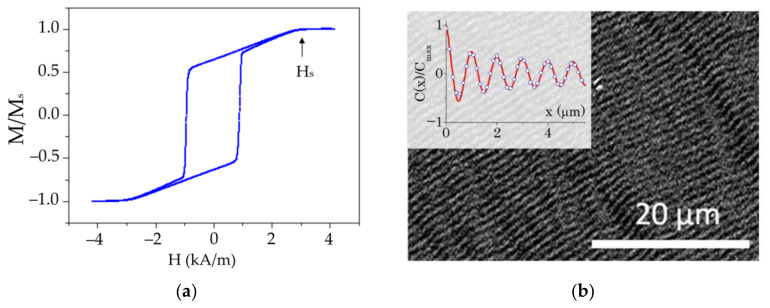
Hysteresis loop of the Py film (500 nm), field applied in the film plane at an angle of 0° to in-plane EA (**a**). The stripe magnetic domain structure in the zero external magnetic field. The inset shows the correlation function in the direction across the stripe structure to the estimate stripe domain width $D_{m}=260\pm 20$ nm (**b**).

In the external magnetic field applied in a film plane, the hysteresis loop of the Py film (500 nm) has the shape typical for the “transcritical” state (Figure 4). The evolution of the micromagnetic state corresponding to the descending branch of the loop is a transition from a quasi-uniform state (containing no closing magnetic domains) above $H_{s}$ to the appearance and development of a stripe structure in the zero external magnetic field [25,27]. The saturation magnetic field $H_{s}$ is conditioned by the magnetic constants and parameters of the film by the following equation [27,28]:

(2) $$1-\frac{H_{s}}{H_{op}}=\frac{1}{2}\sqrt{\frac{2A}{\mu_{0}H_{op}M_{s}}}\cdot L^{-1}{\left[1+\frac{H_{op}}{M_{s}}\right]}^{-1/2}$$

where L is the film thickness, A is the exchange stiffness, $M_{s}$ is the saturation magnetization and $H_{op}$ is the-out-of plane anisotropy field $\left(H_{op}=2K_{op}/M_{s}\right)$. Using $H_{s}=3.18\pm 0.16$ kA/m determined from the hysteresis loops similar to the one shown in Figure 4 and the constants measured for this film (see the supplement $A=(0.90\pm 0.05)\cdot 10^{-11}$ J/m, $M_{s}=800\pm 20$ kA/m) and L=500 nm, one can obtain the value $H_{op}=8.0\pm 0.8$ kA/m.

Another approach for the estimation of the $H_{op}$ value is to use the width of the stripe domains in the “transcritical” state. It was shown in [27] that if the domain width is defined as the size of the area of uniform magnetization, where the transverse magnetization component $\left(m_{z}\right)$ contribution is at least 65% of the maximum value, then such a stripe domain width $\left(D_{m}\right)$ can be described by the Murayama equation [27,29]:

(3) $$D_{m}=\sqrt{L}\cdot {\left(A(K_{op}+\frac{\mu_{0}M_{s}^{2}}{2})/8\mu_{0}K_{op}M_{s}^{2}\right)}^{1/4}$$

Using Kerr image processing (Figure 4b and inset) $D_{m}=260\pm 20$ nm was determined. Solving Equation (2) with the same constants (A and $M_{s}$) that were used for solving Equation (2), the estimated $D_{m}$ and L=500 nm provides the value $K_{op}=(5.2\pm 1.6)\cdot 10^{3}$$\frac{J}{m^{3}}$ or $H_{op}=10\pm 3$ kA/m. Note that the confidence interval of this estimate is consistent with the estimate from the field $H_{s}$ and Equation (2), although the experimental error associated with this method is much higher.

**Figure 5:**
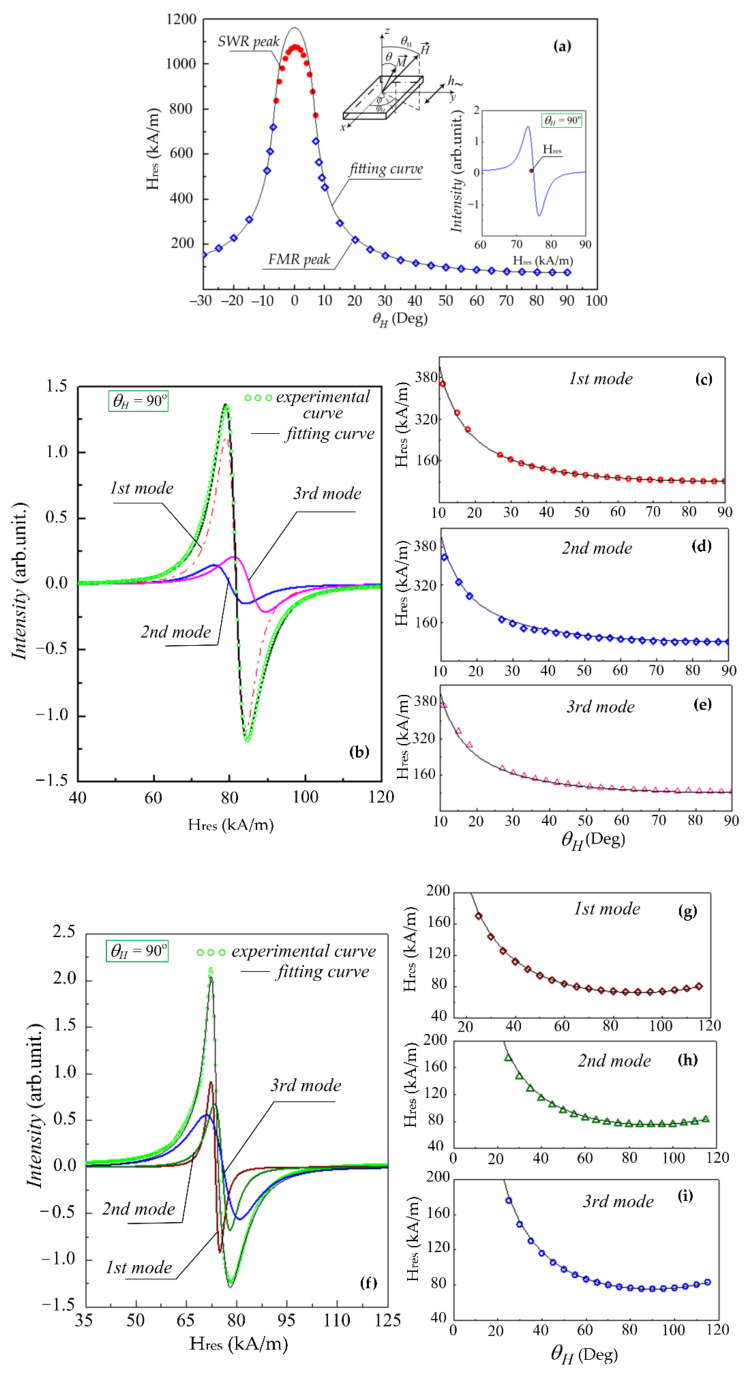
The examples of the microwave spectra of the one-layer films with the thickness 100 nm (inset to Figure (**a**)) and 500 nm (**f**), and the multilayer film (Fe20Ni80/Cu)5 (**b**) measured at $\vartheta_{H}$ = 90°. The solid lines demonstrate the fitting curves of the angular dependences of the resonance fields of the uniform modes fitted according to the Smit–Beljers. The resonance field of the single mode for the 100 nm film (**a**) and the resonance fields of the individual modes for the multilayer film (**c**–**e**) and for the 500 nm film (**g**–**i**) obtained from the fitting of the experimental curve are shown by different symbols.

When the field is oriented out of the film plane, the ferromagnetic resonance field changes in a wide range due to the high contribution to the magnetic anisotropy constant from the film shape $(-\frac{1}{2}\mu_{0}M_{s}^{2}$. In this case, the film magnetization $M_{s}$ is determined both by the Zeeman energy and the value of the magnetic anisotropy constant $K_{op}-\frac{1}{2}\mu_{0}M_{s}^{2}$, ($K_{op}$ is the out of plane or perpendicular anisotropy constant). These terms contribute to the angular dependence of the resonant field in a different way making it possible to estimate $K_{op}$ and $M_{s}$ separately using the Smit–Beljers approach [26]. Thus, both in-plane and out-of-plane anisotropy are present in one film sample. The axis of the out-of-plane rotation was chosen to coincide with the in-plane EA to exclude the influence of the in-plane magnetic anisotropy on the out-of-plane angle dependence of the resonance field. The characteristic angles that are determined by the theoretical expressions for the resonant field and are controlled in the experiment are shown in Figure 5. Thus, for the measurements with the out-of-plane oriented external field, the angle $\phi_{H}$ was chosen as $\phi_{H}=0{}^{\circ}$ to easily study the out of plane magnetic anisotropy.

For the Py film (100 nm), the FMR spectrum (Figure 5a) in the range ϑ of 7° to 90° is well described by a single Lorentzian mode. Furthermore, this mode corresponds to a uniform precession of the magnetization. In the range (−7°÷7°) no uniform oscillation modes of magnetization are excited and several peaks of spin-wave resonance are observed. Figure 5a shows the resonance fields corresponding to the uniform and the 1st spin wave bulk mode. Fitting the angular dependence of the resonance field for the uniform mode using the Smit–Beljers approach [26] for the Py (100 nm) film, gives the following fitting parameters $M_{s}=880\pm 10$ kA/m, $H_{op}=8.0\pm 0.8$ kA/m (see also Table 1). For the Py film (500 nm), the shape of the FMR spectrum is not described by one Lorentzian. However, it can be satisfactorily described by the sum of at least three Lorentzian functions. The multiplicity of the peaks here may be interpreted as the manifestation of inhomogeneity in the thick film, and the peaks in this case are supposed to be associated with some naturally formed layers with the given absorption. It is impossible to specify exactly what these layers are and at what depth each of them is located within the framework of this approach alone. Even so, the separate processing of the data related to these composite peaks can be considered as a useful way of characterizing the film inhomogeneity over the thickness. These layers are apparently the result of the inhomogeneity of elastic deformations over the film thickness. It is hard to observe such layering using transmission electron microscopy. Because of this, we see some value of the proposed approaches using FMR. The issue of this inhomogeneity is apparently related to the film growth mechanism, since it usually determines the inhomogeneity of elastic deformations in deposited films. For example, residual strains in the films can be distributed over the thickness in such a way that compressive stresses are replaced by tensile stresses. A change in the sign of the deformation should lead to a change in the sign of the stress-induced magnetic anisotropy field. The negative anisotropy field in the multilayer sample (Table 1) corresponds to the easy-plane anisotropy. Note that negative H_op_ is observed only in the multilayer sample, where there are more sources of internal deformations due to a larger number of interfaces. The range of angles in which the individual peaks are considered as the result of a uniform precession of the magnetization is $\vartheta_{H}$ of 7° to 90°. The results of fitting of the angular dependence of each peak are given in Table 1. In the multilayered film (Fe_20_Ni_80_/Cu)_5_ with the Py layer thickness of 100 nm, we also observe a non-Lorentzian shaped line, which can also be successfully described as the sum of three Lorentzian peaks. The saturation magnetization and the perpendicular magnetic anisotropy field are determined for each of the three modes and are given in Table 1.

According to Table 1, the single-layer Py film (100 nm) shows more of the uniform microwave response (only one peak) and a wider range of angles (from 7 to 90 up to 90°) of the uniform precession mode than the Py film of 500 nm (from 25 to 90°). The range of the angles is important as a parameter of the sensor element operating at super high frequencies, because the excitation of inhomogeneous precession modes will inevitably lead to many peaks in the film response. In the multilayered film, this range is closer to that of the single-layered film (from 11 to 90°), which indicates the advantages of the multilayer film sensor design.

The deviations of the peak from the Lorentzian shape can be also viewed as a measure of the inhomogeneity of the microwave response [30,31,32]. Let us summarize and discuss the observations concerning it. For the single-layered thin film (Py of 100 nm), a single Lorentz peak is observed: the deviation is negligibly small. For the single-layered Py film of 500 nm the deviation is the largest. For the multilayered structure (with the total thickness of 500 nm) this deviation is much lower than the one observed for the single-layered Py film (500 nm), although it somewhat exceeds the deviation observed for the single-layer film (100 nm). The fields $H_{op}$ in the thick film are close to those for the single-layered Py film (100 nm), and the average of the fields over three components coincides with it (Table 1). This is in agreement with the conclusion (see the analysis for Figure 1) that the constant (or field) of the perpendicular magnetic anisotropy of single-layered films is, in general, uniform over the thickness and, therefore, does not change with the thickness. In addition, the field $H_{op}$ for the Py (100 nm) film coincides with the estimate made from the hysteresis loop of the Py film (500 nm) in the “transcritical” state. These observations reveal additional advantages of the multilayered design for GMI sensors, discussed in a number of previous works [33,34,35].

## 4. Conclusions

The characterization of magnetic anisotropy using magnetization curves and the ferromagnetic resonance techniques of single-layered and multilayered thin film structures based on a permalloy for magnetic field sensors was performed. It was demonstrated that the proposed approaches allowed for not only the characterization of magnetic anisotropy in the plane and perpendicular to the plane of the thin films, but also the study of their inhomogeneity, which is important in the magnetic film sensor design. It is shown that the quantitative data from the direct integral and local measurements of magnetic anisotropy are consistent with the direct and indirect estimates based on the magnetization curve processing. The example of estimating the perpendicular magnetic anisotropy constant from the width of stripe domains in a film in the supercritical state is provided. The average in-plane magnetic anisotropy of the single-layered Py (50, 100 and 500 nm) films prepared by magnetron sputtering onto a Corning glass is uniform through the thickness of the single-layer film. The inhomogeneity of the perpendicular anisotropy field for the 500 nm film is greater than that for the 100 nm film, and the inhomogeneity of the multilayer film (Fe_20_Ni_80_ (100 nm)/Cu (3 nm))_5_ is greater than that for the single-layer of the approximately same thickness.

## Figures and Tables

**Table 1:** The parameters of the single-layered films Fe_20_Ni_80_ (100 nm and 500 nm) and multilayered film (Fe_20_Ni_80_/Cu)_5_ with the Fe_20_Ni_80_ layer thickness of 100 nm, determined from the angular dependences of the FMR.

	M_eff_, kA/m	H_op_, kA/m
Fe_20_Ni_80_ single-layer film of 100 nm	880	8.0
Fe_20_Ni_80_ single-layer film of 500 nm	924	11.9
	894	0
	890	4.0
multilayer (Fe_20_Ni_80_/Cu)_5_ with Fe_20_Ni_80_ layer thickness of 100 nm	758	0
	740	15.9
	790	−35.8

## Data Availability

Data available from the corresponding author upon reasonable request.
